# An unexpected new catalyst promoter: ‘inert’ nitrogen gas!

**DOI:** 10.1093/nsr/nwaa043

**Published:** 2020-04-17

**Authors:** Haichao Liu, Mingyuan He

**Affiliations:** 1 Beijing National Laboratory for Molecular Sciences, College of Chemistry and Molecular Engineering, Peking University, China; 2 Shanghai Key Laboratory of Green Chemistry and Chemical Processes, School of Chemistry and Molecular Engineering, East China Normal University, China

Molecular nitrogen (N_2_) is widely used as a carrier or protective gas in many catalytic reactions because of its chemical inertness and large availability in nature. Up to now, N_2_ has not been recognized as a promoter or an active component to enhance catalytic performance.

However, the textbook description of inert N_2_ has been rewritten in a recent paper published in *Nature Catalysis* by Duan *et al.*, reporting that N_2_ can dramatically promote biomass hydrodeoxygenation (HDO) over ruthenium (Ru)-based catalysts [1]. As exemplified by the HDO of *p*-cresol to toluene, a representative model reaction for upgrading the lignin-rich biomass, the presence of N_2_ led to a 4.3-fold increase in HDO activity over Ru clusters (with an average diameter of 1.2 nm) dispersed on titanium oxide (Ru/TiO_2_) in a batch reactor (at 160°C, 1 bar H_2_ and 6 bar N_2_). Similar promoting effects of N_2_ were also observed when applying other Ru catalysts in the HDO reaction, confirming the ability of N_2_ to unprecedentedly act as a catalytic promoter.

Detailed studies [1], collaboratively carried out by the research groups of Jun Li from Tsinghua University and Edman Tsang and Dermot O’Hare from University of Oxford with complementary expertise in computational catalysis, surface catalysis and biomass conversion, have clarified the mechanism of N_2_ promotion (Fig. 1). *In situ* X-ray absorption near edge structure and *in situ* Fourier-transform infrared spectroscopy show that hydrogenated nitrogen species (N_x_H_y_, *x* = 1, 2, *y* = 1, 2) form on the Ru surface under the aforementioned reaction conditions. Density functional theory calculations show that the activation of N_2_ to NNH and subsequent N_x_H_y_ species follows an *associative reduction mechanism*, which had been previously proposed in theoretical studies on other supported metal clusters under mild conditions [2,3]. These N_x_H_y_ species offer protic hydrogens to assist the removal of –OH groups adsorbed on the Ru surface—a rate-determining step for the HDO of *p*-cresol that consequently lowers the HDO activation energy and enhances its activity (Fig. 1).

**Figure 1.**
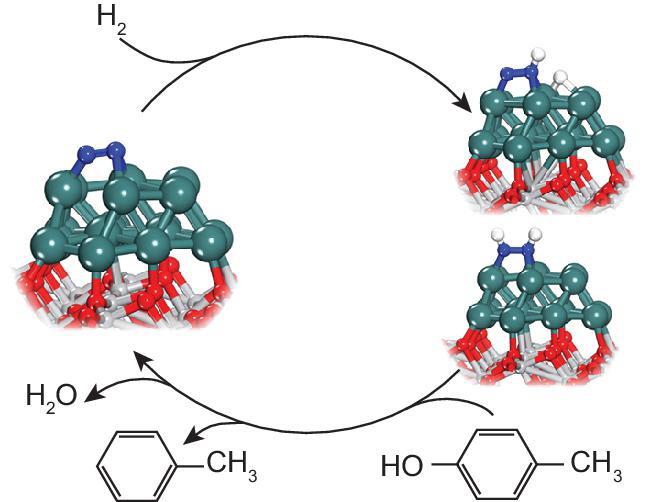
Schematic illustration of the combination of N_2_ activation and HDO reaction on Ru/TiO_2_ (a revised scheme based on Fig. 5 in [1]). N_2_ is converted into NNH and HNNH species on Ru surfaces, which provide protic hydrogen for converting p-cresol into toluene. Color code of the spheres: Ru (green), Ti (light gray), O (red), N (blue), H (white).

This pioneering work provides solid evidence that N_2_ can be activated under less severe conditions via an associative reduction mechanism with the formation of NNH as the initial step. It demonstrates that N_2_ can no longer be considered as an inert carrier gas, since it can act as a catalytic promoter in HDO reactions, which are of practical importance for upgrading biomass-derived oxygenated feedstocks [4–7]. Furthermore, the work provides a potentially general strategy for the rational tuning of catalyst performance under actual reaction conditions.

## References

[1] Duan H , Liu J, Xu M et al. Nat Catal 2019; 2: 1078–87.

[2] Ma X , Liu J-C, Xiao H et al. J Am Chem Soc 2018; 140: 46–9. 29244491 10.1021/jacs.7b10354

[3] Liu J-C , Ma X, Li Y et al. Nat Commun 2018; 9: 1610. 29686395 10.1038/s41467-018-03795-8 PMC5913218

[4] Duan H , Dong J, Gu X et al. Nat Commun 2017; 8: 591. 28928359 10.1038/s41467-017-00596-3 PMC5605710

[5] Liu G , Robertson A, Li M et al. Nat Chem 2017; 9: 810–6. 28754945 10.1038/nchem.2740

[6] Sun Q , Wang S, Liu H. ACS Catal 2017; 7: 4265–75.

[7] Sun Q , Wang S, Liu H. ACS Catal 2019; 9: 11413–25.

